# Screening herbal extracts as biostimulant to increase germination, plant growth and secondary metabolite production in wheatgrass

**DOI:** 10.1038/s41598-023-50513-6

**Published:** 2024-01-05

**Authors:** Anjali Yadav, Shachi Singh, Vidushi Yadav

**Affiliations:** 1 Department of Botany, MMV, Banaras Hindu University, Varanasi, UP 221005 India; 2 Bioinformatics, MMV, Banaras Hindu University, Varanasi, UP 221005 India

**Keywords:** Chemical biology, Plant sciences

## Abstract

Recently it has been recognized that herbal plants contain endogenous molecules with biostimulant properties, capable of inducing morphological and biochemical changes in crop plants. Therefore, the present experiment was conducted to screen herbal samples for their plant growth promoting properties. Twenty-five herbal extracts were tested for their biostimulating activity on wheat crop (*Triticum aestivum*) through seed priming. Morphological parameters chosen for evaluation include: percent seed germination, length and weight of seedling, wheat grass length and biomass. Biochemical parameters include: total phenolic and flavonoid, enzymatic activity of catalase and phenylalanine ammonium lyase and antioxidant activity. Results indicated an increase in the tested parameters by the extracts, however the biostimulant property varied between the selected herbal samples. Some of the samples, such as *Phyllanthus emblica, Plumbago zeylanica, Catharanthus roseus* and *Baccopa monnieri,* were highly effective in inducing plant growth promoting parameters. Principal component analysis was performed and herbal samples were grouped into categories based on their activity.

## Introduction

It is indisputable that wheat is an economically significant crop contributing to both human and animal diets. It is a significant source of calories and also contain a variety of nutrients, including phytochemicals, vitamins and dietary fibres that are required for maintaining a healthy body^1^. Along with wheat grains, wheatgrass has long been a staple of Indian culture and is renowned for its exceptional medicinal qualities. It is a vegetable that is picked before the plant blooms^2^. Regular consumption of wheatgrass extract enhances digestion and improves overall health^3–5^.

Priming is the term for seed energising procedures carried out before germination^6^. Seed priming increases germination speed and uniformity while also causing several biochemical changes that are essential for breaking dormancy, mobilising or hydrolysing seed stores, activating enzymes and promoting plant growth and development^7,8^. To enhance seed germination and plant development, commercial growth regulators have been utilised as priming agents. Exogenous biostimulants have successfully benefited crop plants, such as fruits, vegetables cereals and legumes for better growth, phytochemical, crop yield and quality^9–11^.

It has been reported that some plant compounds influence crop growth and yield. Seed priming with sunflower (*Helianthus annuus* L*.*)^12^, moringa (*Moringa oleifera* Lam*.*)^13^ and sorghum (*Sorghum bicolor* L.)^14^ leaf extracts enhanced the emergence rate, seedling growth, and development of maize (*Zea mays* L.) and wheat (*Triticum aestivum* L.) crop. Zeatin and other plant growth boosters were found in abundance in the extract of moringa leaves^15^, whereas phenolic acids, such as vanillic, coumaric, ferulic and hydroxybenzoic acids were identified in sorghum extract as biostimulating agents^16^. Exogenous application of aqueous garlic extracts on eggplant and pepper seedlings improved growth and phytochemical content of the treated plants^17^. Leaf extracts from aloe (*Aloe barbadensis* L.) and heena (*Lawsonia inermis* L.) improved the growth and chemical composition of *Schefflera arboricola* Hayata (umbrella plant)^18^. Oak bark extract improved colour, polyphenols contents, as well as aroma of grapevines. *Glycyrrhiza glabra* L. (Licorice) root extract has been shown to improve growth and yield of beans. Because of the widespread applicability of natural extracts in modulating plant growth and development, the present study was set up to assess the potential effects of some herbal extracts on the germination process as well as their contribution to the accumulation of bioactive compounds in wheatgrass.

## Materials and methods

### Experimental setup

All methods used in the work were carried out in accordance with relevant guidelines^25^. The studies were conducted in the laboratory of Banaras Hindu University (BHU) Varanasi. Wheat was cultivated in a plant growth chamber with constant humidity (70%) and temperature (25 °C ± 2 °C), while maintaining 16 h of light and 8 h of darkness throughout the study.

### Herbal extract preparation

Herbal extracts were obtained from twenty-five species of herbal plants (Table 1). Stock solutions were prepared using plant tissues, primarily leaves, by homogenising 0.2 g of the fresh tissue in 15 ml of distilled water with the help of pestle and mortar. The extract obtained was immediately filtered through Whatmann filter paper and different dilutions were prepared by taking 2 ml (Solution A), 4 ml (Solution B), and 8 ml (Solution C) of stock solution and achieving a final volume of 20 ml by adding distilled water.

**Table 1:** Basic information of selected herbal plants.

Serial number	Botanical name	Common name	English name	Family	Selected part
1	*Withania somnifera* L.	Ashwagandha	Indian Ginseng	Solanaceae	Leaf
2	*Curcuma longa* L.	Haldi	Turmeric	Zingiberaceae	Leaf
3	*Mentha piperita* L.	Mentha	Mint	Lamiaceae	Leaf
4	*Plumbago zeylanica* L.	Chitrak	Lead war	Plumbaginaceae	Leaf
5	*Catharanthus roseus* L.	Sadabahar	Periwinkle	Apocynaceae	Leaf
6	*Ficus religiosa* L.	Pipal	Pipal	Moraceae	Leaf
7	*Syzygium aromaticum* L.	Laung	Cloves	Myrtaceae	Flower bud
8	*Cymbopogon citratus* L.	Lemon grass	Cymbopogon citratus	Poaceae	Leaf
9	*Ocimum tenuiflorum* L.	Tulsi	Holy basil	Lamiaceae	Leaf
10	*Azadirachta indica* A.	Neem	Margosa	Meliaceae	Leaf
11	*Nyctanthes arbortristis* L.	Parijat	Night Jasmine	Oleaceae	Leaf
12	*Bacopa monnieri* L.	Brahmi	Water hyssop	Scrophulariaceae	Leaf
13	*Jasminum grandiflorum* L.	Chameli	Jasmine	Oleaceae	Leaf
14	*Asparagus racemosus* L.	Stavar	Stavar	Asparagaceae	Leaf
15	*Cinnamomum camphora* L.	Kapoor	Camphor	Lauraceae	Leaf
16	*Tagetes erecta* L.	Geda	Marigold	Asteraceae	Leaf
17	*Eclipta prostrata* L.	Bhrigraj	False daisy	Asteraceae	Leaf
18	*Rauvolfia serpentina* L.	Raulfia	Devil peppers	Apocynaceae	Leaf
19	*Helicteres isora* L.	Madodfali	The Indian screw	*Sterculiaceae*	Leaf
20	*Pluchea lanceolata* DC	Rasna	Thai ginger	Asteraceae	Leaf
21	*Piper longum* L.	Pipli	Long Pepper	Piperaceae	Leaf
22	*Asclepias physocarpa* E. Mey.	Dauna	Sweet marjoram leaves	*Zingiberaceae*	Leaf
23	*Combretum indicum* L.	Madhurani	Burma agrimony	Combretaceae	Leaf
24	*Phyllanthus emblica* L.	Awala	Indian gooseberry	Phyllanthaceae	Leaf
25	*Tinospora cordifolia* Thunb.	Giloy	Tinospora	Menispermaceae	Leaf

### Seed collection

HVW-243 wheat (*Triticum aestivum* L.) variety, one of the oldest and widely cultivated variety in the eastern Gangetic plains, and containing various nutritional composition was selected as experimental material. HVW-243 was purchased from agricultural farm of Banaras Hindu University Varanasi, Uttar Pradesh.

### Germination test

Seeds were surface sterilised with 2% sodium hypochlorite solution, followed by a thorough rinse with distilled water. Then, seeds were soaked for 8 h at room temperature in different herbal extract concentrations; distilled water serving as the control. Ten seeds were spread on a Petri dish containing double layers of moist filter paper. Petri dishes were kept in complete darkness at room temperature for germination. After germination, seedling morphological parameters were calculated, including seedling length, biomass and percentage germination, according to the following formula.

1 $${\text{GP}} = {\text{GS}}/{\text{TS}} \times 100$$

with GP representing the germination percentage, GS the number of germinated seed, and TS the number of sown seeds. For each germination condition, five replicates of each treatment were performed.

### Pot experiment

Seeds were soaked in herbal extracts as well as in distilled water (control), as described above. Thirty seeds were placed in each pot, containing sterilized soil, and were then allowed to grow for 10 days in a plant growth chamber (conditions described above). Plants were watered with sterile distilled water at an interval of three days. After ten days, plants were harvested and wheatgrass length and fresh weight were calculated.

### Estimation of total phenolic content

The Folin–Ciocalteau method was used to measure the total phenolic content, according to the methods described in the literature^19^. One gram of dry biomass of wheat leaves was taken and extracted with 10 ml of 70% methanol. A test tube containing about 0.5 ml of 70% methanolic extract and 0.5 ml of methanol were taken and 300 µl of 50% diluted Folin–Ciocalteau's phenol reagent was added. The reaction mixture was left at room temperature for 10 min in the dark, after which twenty percent aqueous sodium carbonate solution was added. Distilled water was then added to keep the sample's final volume at 3 ml. At 650 nm, the absorbance of the reaction mixture was measured after standing for 60 min at room temperature. As a control, a standard curve was created using gallic acid at various concentrations.

### Estimation of total flavonoid

The total flavonoid content of wheatgrass was measured using reported protocol^20^. One ml of seventy percent of methanolic extract was combined with 1 ml of pure methanol and then 1 ml of 2% AlCl_3_ was added. This combination was incubated for 1 h at room temperature, after which absorbance was measured at 420 nm. In order to prepare a conventional graph, Rutin was utilised as standard.

### Photosynthetic pigments

To estimate the photosynthetic pigments, the method of Takshak and Agrawal 2018^21^ was applied with slight modification. Wheatgrass powder was homogenised in acetone and centrifuged at 5000 rpm for five minutes to extract photosynthetic pigments. Supernatant was collected and the absorbance was measured at 480, 510, 645, and 663 nm to estimate the presence of carotenoids, chlorophyll b (Chl b) and chlorophyll a (Chl a) using the following formulas:

2 $${\text{Chlorophyll a }}\left( {{\text{mg g}}^{{ - {1}}} {\text{FW}}} \right) = \left( {{12}.{\text{3OD}}_{{{663}}} - \, 0.{\text{86OD}}_{{{645}}} } \right) \times {\text{V}}/\left( {{1}000 \times {\text{W}}} \right)$$

3 $${\text{Chlorophyll b }}\left( {{\text{mg g}}^{{ - {1}}} {\text{FW}}} \right) = \left( {{19}.{\text{6OD}}_{{{645}}} - { 3}.{\text{6OD}}_{{{663}}} } \right) \times {\text{V}}/\left( {{1}000 \times {\text{W}}} \right)$$

4 $${\text{Total chlorophyll }}\left( {{\text{mg g}}^{{ - {1}}} {\text{FW}}} \right) = {\text{Chlorophyll a}} + {\text{Chlorophyll b}}$$

5 $${\text{Carotenoids }}\left( {{\text{mg g}}^{{ - {1}}} {\text{FW}}} \right) = \left( {{7}.{\text{6OD}}_{{{48}0}} - { 1}.{\text{49OD}}_{{{51}0}} } \right) \times {\text{V}}/\left( {{1}000 \times {\text{W}}} \right)$$

V is the volume of the extract (ml) and W is the fresh weight of the sample (g).

### 2,2-Diphenyl-1-picrythydrazyl radical assay (DPPH)

The wheatgrass samples were homogenised in 70% methanol and then centrifuged at 5000 rpm for five minutes. The supernatant was collected and used for the measurement of antioxidant activity by DPPH radical scavenging assay^22,23^. In a test tube, 0.5 ml of extract was taken and diluted with 2.5 ml of methanol. The sample mixture was combined with 1 ml of 0.004% DPPH solution and incubated for 30 min in the dark. Ascorbic acid was used as standard. The absorbance was measured at 517 nm and the proportion of antioxidant activity was determined using the following formula:

6 $${\text{Radical scavenging activity}} = {\text{AC}} - {\text{AS}} \times {1}00/{\text{AC}}$$

AC representing absorbance of control and AS representing absorbance of sample. Methanolic wheatgrass extract was the sample and 3 ml methanol were used as control.

### Ferric- reducing antioxidant power assay (FRAP)

The FRAP assay was carried out using Guo methodology^24^. Approximately 0.5 ml of extract was taken and diluted with 0.5 ml of methanol, followed by the addition of 2 ml of FRAP reagent. It was incubated for 15 min at 37 °C, after which absorbance was calculated at 593 nm. By comparing antioxidant activity to ascorbic acid standard solutions, antioxidant activity of the extract was estimated. Fresh FRAP reagent was prepared by combining 2.5 ml of 10 mmol/l 2,4,6-Tripyridy-s-triazine (TPTZ) produced in 40 mmol/l HCl with 2.5 ml of 20 mmol/l FeCl_3_ (prepared in 25 ml of 0.3 mol/L acetate buffer, pH 3.6).

### Peroxidase (POD) activity

Using a chilled mortar and pestle, 2 g of fresh wheatgrass was homogenised in 2 ml of 0.1 M cold phosphate buffer (7 pH). Supernatant of sample was taken and enzyme activity was calculated using method described in literature^25^. A cuvette was filled with 3 ml of 0.1 M phosphate buffer (pH 7.0), 0.05 ml of 4% guaiacol and 0.1 ml of extract and was thoroughly mixed. Spectrophotometer was set to 436 nm, the cuvette was placed and 0.03 ml of 30% H_2_O_2_ was added. Time taken to raise the absorbance by 0.1 was noted. The control was phosphate buffer with guaiacol solution.

### Phenylalanine ammonium-lyase (PAL) activity

The quantity of cinnamic acid produced by the enzyme's reaction was used to assess the activity of PAL enzyme^26^. The reaction mixture contained 0.5 ml of crude extract along with 1 ml of 10 mM Phenylalanine, prepared in 0.1 M Tris–HCl buffer of pH 8.5. After being allowed to continue for 30 min at room temperature, the reaction was halted by adding 0.5 ml of 2 N HCl. The absorbance was measured at 290 nm and a typical graph showing the relationship between absorbance and cinnamic acid content was created. The nmole of cinnamic acid generated during the deamination of l-phenylalanine under normal conditions is comparable to one unit of PAL activity.

### Statistical analysis

Each herbal extract treatment had five repetitions, 10 seeds were taken for the germination test and 30 seeds were taken for the pot experiment for each replicate. All tests were carried out twice. The means and standard error (SE) are displayed as error bars on the graph. The effects of the herbal extract on the biochemical, morphological and yield features of wheatgrass were analysed using a one-way univariate analysis of variance (ANOVA). Following the one-way ANOVA, Duncan's multiple range tests were run as post hoc analyses for various parameters. The SPSS statistical package (SPSS Inc., version 16.0) was used for all statistical calculations. PCA analysis was performed by using regression analysis, varimax rotation, and correlation matrix. IBM SPSS (SPSS Inc., version 16.0), Sigmaplot, and Minitab were used for statistical analyses and graph preparation.

## Result and discussion

**Figure 1:**
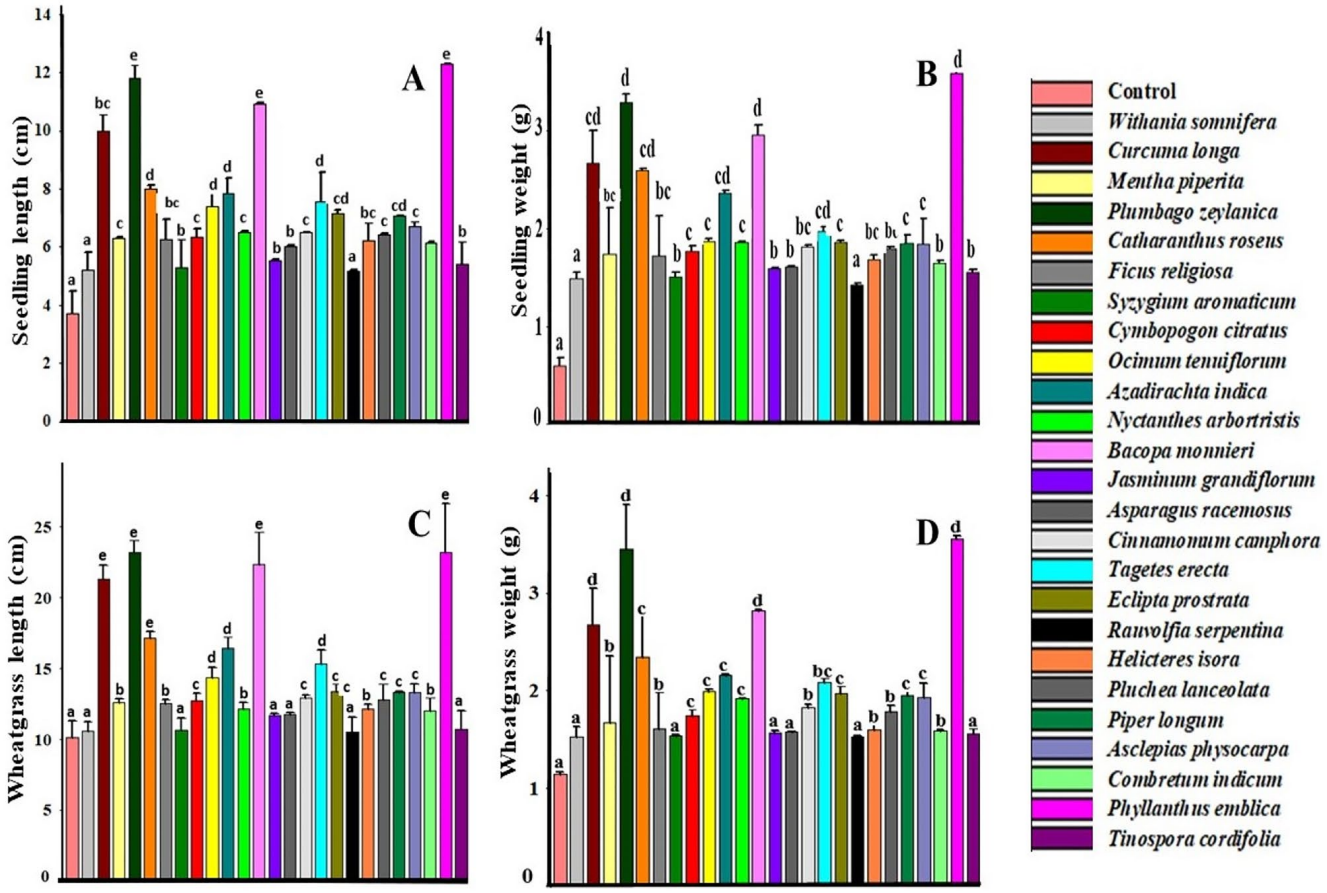
Effect of different herbal extract treatment on morphological parameters of wheat sprouts and wheatgrass (**A**) Seedling length, (**B**) Seedling weight (**C**) Wheatgrass length (**D**) Wheatgrass weight. Values are the means ± standard deviation at significance difference p < 0.05 (different superscripts, a, b, c, d e, bc, cd shows significantly different values).

**Figure 2:**
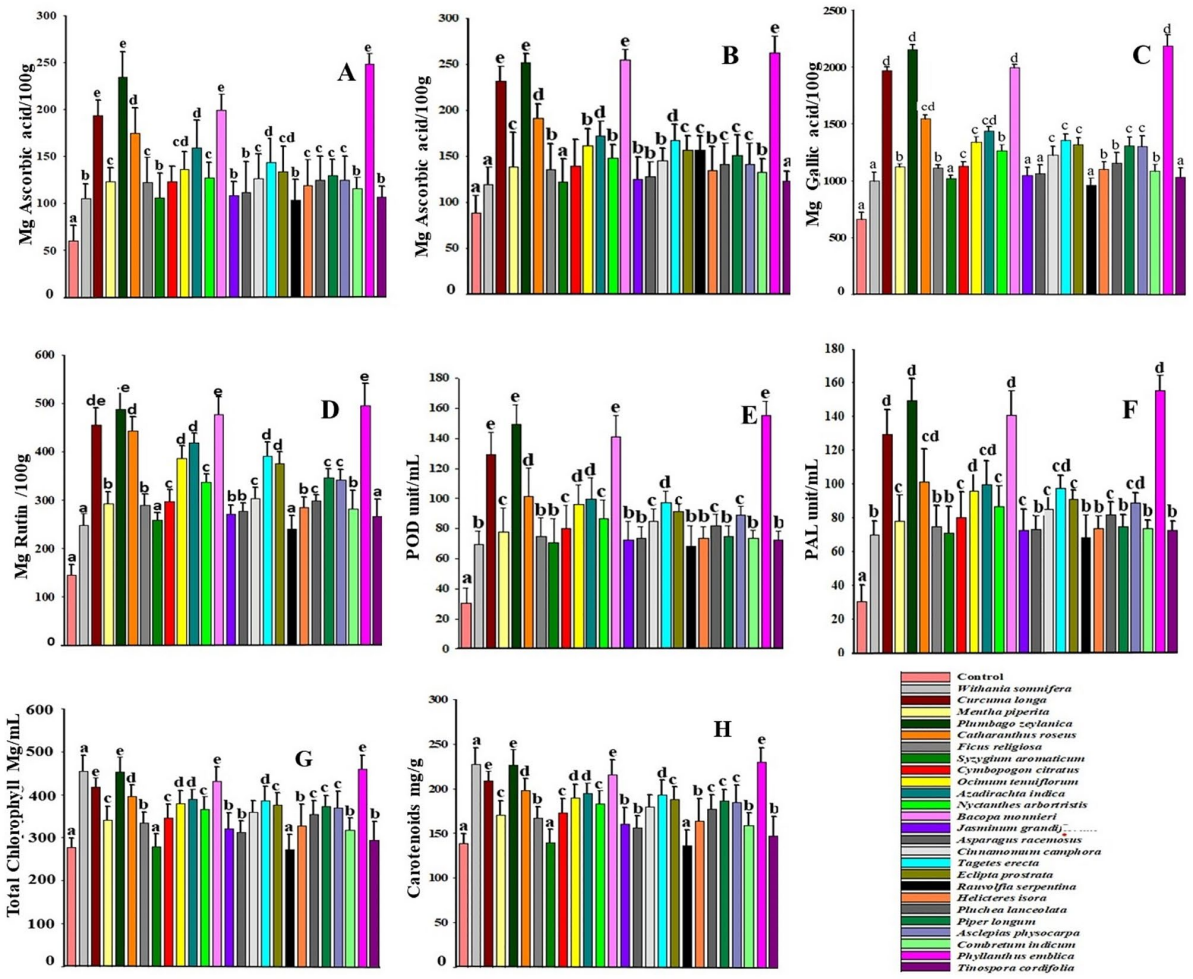
Effect of different herbal extract treatment on biochemical parameters of wheatgrass. (**A**) DPPH assay, (**B**) FRAP assay, (**C**) Total phenolic, (**D**) Total flavonoids, (**E**) POD activity, (**F**) PAL activity, (**G**) Total chlorophyll and (**H**) Total carotenoid. Data are representative of five replicates. Values are the means ± standard deviation, at significance difference p < 0.05 (different superscripts, a, b, c, d e, bc, cd shows significantly different values).

Twenty-five herbal samples with three dilutions of each extract were tested for their plant growth promoting activity. Effect of the treatments on morphological and biochemical parameters of wheat sprout and wheatgrass is shown in Tables 2 and 3. Data analysis revealed an increase in the tested parameters by the treatments, however, the effect varied with the type of herbal extract and its dilution. Figures 1 and 2 displays a comparative assessment of biostimulant activity of all the 25 herbal samples, taking best dilution of each extract for graph preparation. A detailed analysis of the experimental result is presented below.

**Table 2:** Morphological parameters measured after herbal extract treatment: Percentage germination (%), Seedling length (cm), Seedling weight (g), wheatgrass length (cm) and wheatgrass weight (g). A, B and C are dilutions of extracts indicating high, moderate and low concentrations. Letters in bold indicate dilutions with the highest activity. Data are representative of five replicates. Values are the means ± standard deviation.

	Concentration	Percentage germination	Seedling length (cm)	Seedling weight (g)	Wheatgrass length (cm)	Wheatgrass weight (g)
Control		90 ± 14.14	3.41 ± 0.48	0.60 ± 0.08	10.12 ± 1.15	1.15 ± 0.02
* W. somnifera*	A	100 ± 0.00	5.02 ± 0.48	0.98 ± 0.05	10.42 ± 0.43	1.25 ± 0.01
	**B**	**100 ± 0.00**	**5.21 ± 0.59**	**1.49 ± 0.06**	**10.57 ± 0.65**	**1.53 ± 0.10**
	C	100 ± 0.00	4.34 ± 0.47	0.85 ± 0.03	10.45 ± 1.45	1.49 ± 0.02
* C. longa*	A	100 ± 0.00	6.81 ± 0.64	1.17 ± 0.65	17.35 ± 0.93	2.26 ± 0.18
	**B**	**100 ± 0.00**	**9.98 ± 0.58**	**2.67 ± 0.33**	**21.33 ± 0.92**	**2.68 ± 0.37**
	C	80 ± 0.00	4.33 ± 0.44	0.96 ± 0.76	15.25 ± 0.75	1.65 ± 0.14
* M. piperita*	A	100 ± 0.00	5.54 ± 0.27	0.98 ± 0.74	12.45 ± 0.36	1.41 ± 0.55
	B	100 ± 0.00	4.85 ± 1.02	0.57 ± 0.54	11.37 ± 0.45	0.97 ± 0.39
	**C**	**100 ± 0.00**	**6.29 ± 0.06**	**1.74 ± 0.47**	**12.58 ± 0.26**	**1.67 ± 0.69**
* P. zeylanica*	A	100 ± 0.00	8.36 ± 0.65	1.65 ± 0.02	17.95 ± 0.63	2.65 ± 0.18
	**B**	**100 ± 0.00**	**11.81 ± 0.44**	**3.29 ± 0.08**	**23.15 ± 0.86**	**3.45 ± 0.45**
	C	100 ± 0.00	9.03 ± 0.74	2.81 ± 0.04	19.75 ± 0.86	3.25 ± 0.83
* C. roseus*	A	100 ± 0.00	7.19 ± 0.04	1.88 ± 0.04	16.45 ± 0.29	1.95 ± 0.69
	**B**	**100 ± 0.00**	**7.98 ± 0.15**	**2.59 ± 0.02**	**17.16 ± 0.47**	**2.34 ± 0.43**
	C	100 ± 0.00	6.52 ± 1.02	1.69 ± 0.03	15.25 ± 0.95	1.78 ± 0.87
* F. religiosa*	A	100 ± 0.00	5.91 ± 0.62	0.99 ± 0.07	11.95 ± 0.96	1.38 ± 0.28
	B	100 ± 0.00	6.14 ± 1.29	1.25 ± 0.01	12.49 ± 0.61	1.51 ± 0.63
	**C**	**100 ± 0.00**	**6.26 ± 0.67**	**1.72 ± 0.41**	**12.55 ± 0.25**	**1.61 ± 0.37**
* S. aromaticum*	A	100 ± 0.00	4.93 ± 0.63	0.93 ± 0.02	10.41 ± 0.72	1.36 ± -.16
	**B**	**100 ± 0.00**	**5.29 ± 0.95**	**1.51 ± 0.04**	**10.65 ± 0.86**	**1.54 ± 0.01**
	C	100 ± 0.00	5.16 ± 0.74	1.35 ± 0.14	10.55 ± 0.41	1.45 ± 0.05
* C. citratus*	A	100 ± 0.00	5.06 ± 0.49	0.97 ± 0.01	12.15 ± 0.72	1.32 ± 0.06
	B	100 ± 0.00	6.22 ± 0.41	1.29 ± 0.02	12.55 ± 0.98	1.54 ± 0.09
	**C**	**100 ± 0.00**	**6.35 ± 0.28**	**1.77 ± 0.05**	**12.75 ± 0.51**	**1.75 ± 0.06**
* O. tenuiflorum*	A	100 ± 0.00	5.72 ± 0.91	0.83 ± 0.02	12.63 ± 0.49	1.36 ± 0.3
	B	100 ± 0.00	6.61 ± 0.59	1.15 ± 0.01	13.54 ± 0.76	1.61 ± 0.01
	**C**	**100 ± 0.00**	**7.39 ± 0.43**	**1.87 ± 0.03**	**14.35 ± 0.72**	**1.99 ± 0.02**
* A. indica*	A	**100 ± 0.00**	**7.85 ± 0.52**	**2.36 ± 0.03**	**16.46 ± 0.78**	**2.16 ± 0.01**
	B	100 ± 0.00	5.61 ± 0.21	1.81 ± 0.01	14.35 ± 0.91	1.75 ± 0.03
	C	100 ± 0.00	4.99 ± 0.06	0.98 ± 0.02	12.15 ± 0.66	1.25 ± 0.02
* N. arbortrotis*	**A**	**100 ± 0.00**	**6.51 ± 0.01**	**1.86 ± 0.01**	**13.0510.15**	**1.92 ± 0.01**
	B	100 ± 0.00	6.14 ± 0.03	1.47 ± 0.02	12.16 ± 0.46	1.82 ± 0.03
	C	80 ± 0.00	6.01 ± 0.02	0.75 ± 0.02	11.51 ± 0.2	1.40 ± 0.02
* B. monnieri*	**A**	**100 ± 0.00**	**10.93 ± 0.03**	**2.95 ± 0.11**	**22.33 ± 2.31**	**2.82 ± 0.01**
	B	100 ± 0.00	8.79 ± 0.04	1.94 ± 0.09	18.65 ± 1.13	1.68 ± 0.02
	C	80 ± 0.00	8.01 ± 0.02	1.74 ± 0.01	16.85 ± 2.1	1.05 ± 0.01
* J. Grandiflorum*	**A**	**100 ± 0.00**	**5.51 ± 0.04**	**1.71 ± 0.01**	**11.66 ± 0.17**	**1.57 ± 0.02**
	B	100 ± 0.00	5.25 ± 0.06	1.43 ± 0.05	11.55 ± 0.61	1.42 ± 0.01
	C	100 ± 0.00	4.53 ± 0.12	0.51 ± 0.02	10.75 ± 0.38	1.25 ± 0.03
* A. racemosus*	A	100 ± 0.00	5.65 ± 0.03	0.89 ± 0.03	11.31 ± 0.21	1.39 ± 0.02
	**B**	**100 ± 0.00**	**5.95 ± 0.01**	**1.44 ± 0.01**	**11.61 ± 0.16**	**1.46 ± 0.03**
	C	100 ± 0.00	6.03 ± 0.02	1.59 ± 0.01	11.74 ± 0.11	1.58 ± 0.01
* C. camphora*	A	100 ± 0.00	6.19 ± 0.02	0.97 ± 0.01	12.74 ± 1.05	1.73 ± 0.02
	**B**	**100 ± 0.00**	**6.49 ± 0.01**	**1.81 ± 0.02**	**12.93 ± 0.16**	**1.83 ± 0.03**
	C	100 ± 0.00	4.64 ± 0.02	0.57 ± 0.01	12.55 ± 0.25	1.67 ± 0.01
* T. erecta*	**A**	**100 ± 0 .00**	**7.57 ± 1.01**	**1.97 ± 0.05**	**15.31 ± 0.96**	**2.09 ± 0.04**
	B	100 ± 0.00	6.43 ± 0.16	1.62 ± 0.01	14.52 ± 0.61	1.95 ± 0.02
	C	80 ± 0.00	5.41 ± 0.25	0.68 ± 0.02	13.27 ± 0.81	1.67 ± 0.03
* E. prostrata*	**A**	**100 ± 0.00**	**7.15 ± 0.13**	**1.86 ± 0.02**	**13.39 ± 0.52**	**1.97 ± 0.07**
	B	100 ± 0.00	6.57 ± 0.12	1.33 ± 0.01	12.85 ± 1.23	1.62 ± 0.06
	C	100 ± 0.00	4.68 ± 0.01	0.65 ± 0.01	12.45 ± 1.02	1.41 ± 0.02
* R. serpentine*	**A**	**100 ± 0.00**	**5.18 ± 0.02**	**1.42 ± 0.02**	**10.53 ± 1.01**	**1.53 ± 0.01**
	B	100 ± 0.00	4.65 ± 0.01	1.22 ± 0.01	10.45 ± 1.23	1.45 ± 0.01
	C	80 ± 0.00	4.21 ± 0.03	1.15 ± 0.02	10.35 ± 1.31	1.27 ± 0.02
* H. isora*	**A**	**100 ± 0.00**	**6.22 ± 0.61**	**1.68 ± 0.05**	**12.13 ± 0.36**	**1.60 ± 0.05**
	B	100 ± 0.00	5.31 ± 0.38	0.97 ± 0.01	12.05 ± 1.22	1.48 ± 0.07
	C	80 ± 0.00	5.25 ± 0.47	0.65 ± 0.02	11.75 ± 0.79	1.02 ± 0.09
* P. lanceolate*	A	100 ± 0.00	6.36 ± 0.04	0.85 ± 0.01	12.65 ± 1.02	1.40 ± 0.07
	**B**	**100 ± 0.00**	**6.41 ± 0.06**	**1.79 ± 0.02**	**12.81 ± 1.11**	**1.79 ± 0.06**
	C	80 ± 0.00	4.75 ± 0.07	0.61 ± 0.01	12.05 ± 1.02	1.17 ± 0.02
* P. longum*	A	100 ± 0.00	5.98 ± 0.03	0.76 ± 0.01	13.07 ± 0.07	1.78 ± 0.27
	B	**100 ± 0.00**	**7.06 ± 0.01**	**1.85 ± 0.08**	**13.33 ± 0.03**	**1.95 ± 0.06**
	C	100 ± 0.00	5.09 ± 0.02	0.81 ± 0.01	13.15 ± 0.05	1.69 ± 0.82
* A. physocarpa*	A	100 ± 0.00	6.28 ± 0.91	1.38 ± 0.01	12.91 ± 0.03	1.66 ± 0.47
	**B**	**100 ± 0.00**	**6.69 ± 0.16**	**1.84 ± 0.26**	**13.29 ± 0.61**	**1.93 ± 0.15**
	C	80 ± 0.00	6.23 ± 0.75	0.91 ± 0.18	12.55 ± 0.04	1.53 ± 0.21
* C. indicum*	A	100 ± 0.00	6.03 ± 0.05	1.14 ± 0.01	11.82 ± 0.23	1.24 ± 0.01
	**B**	**100 ± 0.00**	**6.14 ± 0.03**	**1.65 ± 0.02**	**12.01 ± 0.89**	**1.59 ± 0.01**
	C	100 ± 0.00	6.10 ± 0.01	1.45 ± 0.02	11.97 ± 1.03	1.39 ± 0.02
* P. emblica*	A	100 ± 0.00	8.63 ± 0.01	2.88 ± 0.02	17.61 ± 1.11	2.36 ± 0.02
	B	100 ± 0.00	10.02 ± 0.03	2.99 ± 0.01	19.35 ± 2.03	2.75 ± 0.01
	**C**	**100 ± 0.00**	**12.28 ± 0.02**	**3.59 ± 0.01**	**23.19 ± 3.45**	**3.55 ± 0.03**
* T. cordifolia*	A	100 ± 0.00	5.16 ± 0.56	1.25 ± 0.01	10.63 ± 1.06	1.47 ± 0.04
	**B**	**100 ± 0.00**	**5.42 ± 0.74**	**1.55 ± 0.03**	**10.71 ± 1.27**	**1.56 ± 0.05**
	C	100 ± 0.00	5.12 ± 1.00	1.15 ± 0.01	10.41 ± 1.73	1.32 ± 0.12

**Table 3:** Biochemical analysis i.e., phenol (GAE/100 g), flavonoid (Rutin/100 g), peroxidase (unit/ml) phenylalanine ammonia-lyase (unit/ml), DPPH (% inhibition), FRAP (mg ascorbic acid/100 g), chlorophyll (mg/g) and carotenoids (mg/g) of all treated herbal extract. A, B and C are dilutions of extracts indicating high, moderate and low concentrations. Letters in bold indicate dilutions with the highest activity. Data are representative of five replicates. Values are the means ± standard deviation.

	Sample name	DPPH (Mg ascorbic acid/100 g)	FRAP (Mg ascorbic acid/100 g)	Phenol (GAE/100 g)	Flavonoid (Rutin/100 g)	Peroxidase (Unit/ml)	Phenylalanine ammonia-lyase (Unit/ml)	Chlorophyll mg/g	Carotenoids mg/g
Control	C	60.37 ± 16.17	88.95 ± 18.71	659.71 ± 61.17	144.56 ± 21.76	30.36 ± 9.93	21.67 ± 3.98	171.62 ± 14.75	138.38 ± 11.04
* W. somnifera*	A	80.68 ± 19.49	93.25 ± 22.38	734.12 ± 27.72	189.02 ± 22.96	49.85 ± 3.51	42.25 ± 6.63	**346.07 ± 29.12**	**139.54 ± 18.96**
	B	**97.53 ± 27.37**	**102.65 ± 28.39**	768.99 ± 97.20	241.09 ± 16.49	61.52 ± 8.67	48.94 ± 8.63	327.07 ± 16.16	127.09 ± 10.07
	C	104.68 ± 15.63	119.15 ± 19.48	**995.02 ± 76.18**	**247.23 ± 25.41**	**69.63 ± 8.53**	**53.86 ± 9.12**	301.73 ± 18.83	115.41 ± 16.65
* C. longa*	A	124.78 ± 29.19	171.75 ± 20.89	827.54 ± 60.05	196.59 ± 17.95	94.42 ± 17.64	71.56 ± 8.25	**493.11 ± 26.57**	**215.12 ± 10.26**
	B	166.92 ± 10.24	180.17 ± 18.39	1208.28 ± 37.39	396.79 ± 13.22	78.53 ± 16.51	64.55 ± 9.94	459.89 ± 18.69	198.14 ± 16.42
	C	**193.37 ± 16.32**	**231.81 ± 16.07**	**1969.55 ± 33.39**	**455.32 ± 35.25**	**129.39 ± 14.71**	**89.17 ± 8.28**	391.49 ± 16.65	171.88 ± 17.91
* M. piperita*	A	91.36 ± 25.81	101.83 ± 24.35	926.33 ± 57.22	189.61 ± 48.94	52.82 ± 16.03	45.91 ± 13.08	**363.16 ± 27.29**	**172.69 ± 16.41**
	B	123.19 ± 14.47	138.58 ± 37.75	1120.46 ± 29.14	292.04 ± 25.34	66.73 ± 29.25	55.07 ± 24.13	355.43 ± 39.08	158.75 ± 25.48
	C	111.64 ± 24.25	125.12 ± 17.40	1048.78 ± 67.35	247.53 ± 55.34	77.91 ± 15.64	61.84 ± 27.30	331.95 ± 24.53	132.04 ± 15.85
* P. zeylanica*	A	127.75 ± 21.14	146.32 ± 16.15	921.59 ± 60.96	195.35 ± 19.83	92.25 ± 9.51	75.85 ± 12.53	**514.24 ± 25.87**	**226.37 ± 17.81**
	B	157.19 ± 15.67	170.59 ± 17.88	1192.47 ± 54.65	306.95 ± 17.39	115.45 ± 14.34	96.22 ± 15.36	488.65 ± 15.81	204.23 ± 13.56
	C	**234.34 ± 27.21**	**251.68 ± 19.91**	**2154.36 ± 44.13**	**487.78 ± 43.32**	**149.54 ± 12.74**	**125.75 ± 9.04**	414.77 ± 11.21	197.95 ± 13.61
* C. roseus*	A	94.45 ± 19.97	116.92 ± 14.91	862.48 ± 29.41	261.85 ± 17.19	66.64 ± 18.54	54.05 ± 15.53	**426.79 ± 29.18**	**209.06 ± 13.29**
	B	116.65 ± 17.77	136.71 ± 13.89	938.95 ± 16.65	326.25 ± 18.82	89.75 ± 7.69	67.43 ± 17.86	407.51 ± 25.79	174.47 ± 19.75
	C	**174.56 ± 26.83**	**191.28 ± 15.37**	**1545.26 ± 30.76**	**443.61 ± 28.82**	**101.41 ± 19.18**	**85.36 ± 9.93**	371.56 ± 17.25	163.51 ± 15.93
* F. religiosa*	A	90.95 ± 25.81	108.81 ± 13.66	916.81 ± 36.35	181.75 ± 15.37	56.63 ± 12.51	35.91 ± 9.69	**361.92 ± 13.48**	**169.99 ± 12.79**
	B	101.72 ± 17.86	114.45 ± 23.68	1022.87 ± 47.92	230.65 ± 14.75	68.72 ± 23.57	43.75 ± 16.17	349.65 ± 14.17	150.97 ± 13.97
	C	**122.35 ± 26.75**	**135.95 ± 27.14**	**1114.03 ± 23.75**	**289.71 ± 21.92**	**74.59 ± 12.78**	**59.94 ± 14.04**	330.79 ± 23.17	141.25 ± 23.87
* S. aromaticum*	A	89.59 ± 36.28	99.42 ± 13.82	835.98 ± 58.91	172.38 ± 13.81	54.32 ± 14.91	41.24 ± 14.64	**349.23 ± 15.18**	**149.56 ± 14.73**
	B	**105.75 ± 26.02**	**121.89 ± 25.44**	**1019.39 ± 27.62**	**258.28 ± 16.33**	**70.87 ± 15.85**	**54.72 ± 18.18**	322.83 ± 27.21	122.83 ± 24.52
	C	91.26 ± 17.93	109.25 ± 16.12	933.45 ± 42.86	221.38 ± 17.64	65.65 ± 12.94	49.51 ± 14.28	301.75 ± 16.87	111.35 ± 16.59
* C. citratus*	A	99.14 ± 22.21	106.86 ± 16.34	951.23 ± 32.05	187.61 ± 21.75	49.79 ± 14.73	39.07 ± 13.46	**364.72 ± 17.83**	**176.89 ± 16.46**
	B	115.52 ± 27.47	121.25 ± 11.76	1050.84 ± 32.58	248.93 ± 16.33	69.71 ± 17.73	51.75 ± 18.35	340.95 ± 27.62	162.26 ± 16.67
	C	**123.23 ± 15.91**	**139.48 ± 28.38**	**1129.51 ± 36.01**	**296.54 ± 25.23**	**79.92 ± 15.53**	**63.93 ± 13.71**	321.75 ± 13.17	151.95 ± 18.08
* O. tenuiflorum*	A	91.26 ± 26.35	115.45 ± 19.68	919.12 ± 60.41	189.73 ± 19.02	49.97 ± 16.26	34.21 ± 18.16	**381.77 ± 19.56**	**192.86 ± 14.83**
	B	**135.77 ± 19.36**	**161.15 ± 18.73**	**1339.18 ± 48.26**	**386.53 ± 25.85**	**95.92 ± 13.38**	**72.11 ± 17.27**	361.11 ± 13.84	168.35 ± 15.61
	C	117.65 ± 16.25	146.35 ± 15.68	1111.41 ± 82.69	282.62 ± 23.10	59.42 ± 13.51	41.17 ± 14.21	341.75 ± 15.22	153.65 ± 14.57
* A. indica*	A	97.72 ± 26.51	109.29 ± 15.25	942.69 ± 61.16	190.24 ± 12.41	49.99 ± 14.24	37.28 ± 12.49	**414.75 ± 16.27**	**198.14 ± 12.03**
	B	122.25 ± 21.37	153.85 ± 16.02	1158.63 ± 35.27	370.25 ± 11.92	64.95 ± 13.18	43.05 ± 13.92	391.43 ± 16.38	185.01 ± 14.38
	C	**158.73 ± 29.37**	**172.25 ± 16.27**	**1439.45 ± 37.07**	**417.71 ± 20.03**	**99.92 ± 13.63**	**81.15 ± 14.38**	373.85 ± 16.19	163.56 ± 16.27
* N. arbortrotis*	A	93.24 ± 22.94	101.68 ± 18.41	919.12 ± 72.82	196.21 ± 14.52	42.12 ± 12.49	**90.11 ± 12.41**	**368.93 ± 14.15**	**186.02 ± 15.01**
	B	105.38 ± 17.71	119.64 ± 13.81	1091.98 ± 63.24	240.97 ± 16.69	68.13 ± 13.15	58.17 ± 13.72	351.85 ± 15.27	171.34 ± 16.03
	C	**127.35 ± 15.92**	**147.96 ± 14.92**	**1261.74 ± 58.24**	**337.65 ± 16.27**	**86.85 ± 12.47**	66.61 ± 12.48	338.76 ± 14.02	154.23 ± 13.44
* B. monnieri*	A	129.26 ± 27.44	155.45 ± 29.13	819.11 ± 57.64	269.21 ± 13.51	60.59 ± 8.18	49.21 ± 15.45	**509.31 ± 12.47**	**226.87 ± 17.25**
	B	159.15 ± 39.28	181.58 ± 24.92	1005.94 ± 59.24	368.24 ± 15.43	91.43 ± 16.89	62.81 ± 14.66	487.61 ± 11.37	209.31 ± 14.35
	C	**199.15 ± 17.19**	**254.68 ± 11.31**	**1994.51 ± 27.62**	**476.44 ± 36.49**	**140.81 ± 14.40**	**98.13 ± 9.13**	469.25 ± 18.35	188.15 ± 11.35
* J. Grandiflorum*	A	92.62 ± 13.63	101.28 ± 25.12	747.24 ± 44.54	186.59 ± 15.51	58.85 ± 11.25	44.82 ± 14.48	**356.04 ± 26.51**	**158.89 ± 18.51**
	B	**107.96 ± 15.26**	**125.12 ± 23.45**	**1048.85 ± 66.41**	**271.64 ± 18.37**	**72.64 ± 12.41**	**55.95 ± 4.53**	339.22 ± 14.96	135.89 ± 16.64
	C	99.84 ± 21.25	114.12 ± 25.21	931.03 ± 51.95	237.75 ± 17.43	65.08 ± 7.22	52.15 ± 5.82	311.36 ± 17.62	123.54 ± 10.27
* A. racemosus*	A	99.28 ± 26.89	109.96 ± 18.11	936.46 ± 36.26	195.09 ± 15.52	46.24 ± 9.32	42.08 ± 5.45	**359.22 ± 19.18**	**160.25 ± 14.07**
	B	104.22 ± 34.33	114.31 ± 14.87	947.68 ± 86.61	248.15 ± 19.39	61.32 ± 6.25	48.76 ± 8.54	332.17 ± 15.81	142.25 ± 16.51
	C	**111.51 ± 33.15**	**127.86 ± 15.83**	**1063.51 ± 72.91**	**277.02 ± 17.04**	**73.33 ± 8.11**	**57.62 ± 5.57**	313.77 ± 16.23	131.79 ± 15.92
* C. camphora*	A	86.86 ± 19.48	99.56 ± 26.69	850.26 ± 33.95	199.38 ± 60.04	57.83 ± 8.08	44.62 ± 13.36	**365.61 ± 16.73**	**184.99 ± 13.64**
	B	101.95 ± 28.99	115.75 ± 17.27	1089.35 ± 72.73	268.29 ± 10.11	61.75 ± 6.88	51.75 ± 9.51	344.89 ± 14.41	164.25 ± 14.46
	C	**126.28 ± 26.61**	**145.59 ± 13.21**	**1223.48 ± 80.12**	**302.62 ± 23.46**	**84.94 ± 7.96**	**65.97 ± 15.91**	323.45 ± 16.14	144.38 ± 12.37
* T. erecta*	A	99.56 ± 27.52	106.83 ± 14.81	933.85 ± 61.41	196.06 ± 14.08	58.85 ± 8.16	49.09 ± 16.89	**392.82 ± 16.91**	**194.65 ± 16.85**
	B	117.25 ± 17.69	139.31 ± 18.35	1142.61 ± 75.15	268.75 ± 13.83	63.33 ± 9.21	51.65 ± 17.31	360.83 ± 15.99	181.89 ± 19.01
	C	**143.18 ± 25.63**	**167.05 ± 17.41**	**1356.54 ± 53.96**	**391.32 ± 28.95**	**97.43 ± 7.51**	**75.27 ± 14.07**	348.14 ± 16.07	174.54 ± 16.51
* E. prostrata*	A	82.93 ± 17.91	105.50 ± 25.37	806.18 ± 94.79	198.51 ± 29.52	52.10 ± 7.49	41.60 ± 7.25	**378.91 ± 25.47**	**189.75 ± 14.36**
	B	**133.47 ± 27.24**	**157.16 ± 15.25**	**1318.14 ± 58.03**	**375.83 ± 23.70**	**91.13 ± 5.34**	**69.08 ± 6.35**	369.22 ± 25.36	177.83 ± 17.51
	C	106.96 ± 25.63	135.89 ± 22.65	1273.48 ± 52.71	305.47 ± 13.36	85.08 ± 6.17	62.19 ± 5.25	344.31 ± 25.35	161.91 ± 12.56
*R. serpentine*	A	78.86 ± 12.15	88.83 ± 25.35	751.35 ± 55.02	184.15 ± 19.45	47.37 ± 7.35	39.16 ± 7.13	**345.95 ± 19.16**	**139.14 ± 17.36**
	B	**103.56 ± 22.15**	**115.83 ± 29.46**	**961.04 ± 62.82**	**239.63 ± 28.64**	**68.45 ± 13.45**	**53.21 ± 8.36**	315.40 ± 19.58	123.75 ± 15.18
	C	96.64 ± 27.25	107.12 ± 25.36	921.09 ± 82.51	217.94 ± 12.23	55.83 ± 18.l48	44.13 ± 5.23	298.48 ± 11.48	109.54 ± 17.15
H. isora	A	79.11 ± 15.34	89.33 ± 15.36	836.35 ± 68.09	184.61 ± 19.41	53.33 ± 8.65	44.91 ± 7.35	**361.16 ± 23.85**	**166.96 ± 25.52**
	B	86.34 ± 25.47	101.55 ± 21.38	917.41 ± 72.55	231.03 ± 12.80	62.82 ± 9.63	55.57 ± 6.35	342.65 ± 25.72	141.25 ± 14.56
	C	**118.94 ± 27.46**	**134.59 ± 25.82**	**1101.64 ± 62.34**	**284.13 ± 22.23**	**73.85 ± 7.15**	**59.92 ± 3.84**	331.81 ± 23.58	128.04 ± 17.95
* P. lanceolate*	A	89.53 ± 15.36	91.32 ± 15.36	711.55 ± 62.18	162.85 ± 33.88	67.51 ± 7.47	55.81 ± 15.46	**364.87 ± 25.42**	**182.79 ± 15.73**
	B	117.19 ± 21.74	118.09 ± 15.74	949.74 ± 46.39	179.34 ± 19.51	71.18 ± 8.25	62.37 ± 9.35	340.68 ± 15.45	161.83 ± 13.73
	C	**124.46 ± 25.83**	**141.58 ± 22.47**	**1153.93 ± 91.22**	**299.11 ± 12.43**	**81.94 ± 7.51**	**64.72 ± 7.36**	331.27 ± 18.47	155.17 ± 14.62
* P. longum*	A	86.28 ± 7.25	99.32 ± 15.46	821.55 ± 43.71	188.25 ± 37.01	59.01 ± 7.85	40.31 ± 9.63	**374.96 ± 25.58**	**187.58 ± 12.52**
	B	96.24 ± 11.44	110.59 ± 12.74	1042.49 ± 67.98	216.45 ± 16.14	60.18 ± 9.74	53.12 ± 7.25	361.43 ± 22.63	166.48 ± 14.62
	C	**129.29 ± 17.25**	**151.11 ± 21.57**	**1306.43 ± 74.63**	**345.91 ± 19.21**	**74.59 ± 7.37**	**67.03 ± 7.54**	341.27 ± 19.73	156.42 ± 19.25
* A. physocarpa*	A	89.15 ± 8.47	101.92 ± 15.47	825.55 ± 92.63	186.35 ± 37.12	61.14 ± 15.57	62.85 ± 6.75	**370.79 ± 22.57**	**198.14 ± 18.84**
	B	106.68 ± 7.95	121.71 ± 15.36	1032.49 ± 79.43	246.85 ± 26.51	72.43 ± 9.64	71.20 ± 8.47	354.01 ± 19.64	161.97 ± 18.74
	C	**128.14 ± 11.63**	**150.86 ± 19.46**	**1301.85 ± 91.16**	**341.13 ± 21.47**	**88.85 ± 5.83**	**82.31 ± 7.54**	331.56 ± 17.84	143.26 ± 17.74
* C. indicum*	A	80.95 ± 13.65	98.58 ± 12.57	842.82 ± 57.85	191.03 ± 26.46	49.13 ± 15.36	31.09 ± 8.58	**359.22 ± 25.25**	**163.51 ± 13.64**
	B	91.72 ± 16.84	101.90 ± 9.58	960.31 ± 78.37	235.65 ± 86.25	63.72 ± 9.46	40.51 ± 7.45	334.23 ± 16.55	148.47 ± 16.73
	C	**115.64 ± 12.63**	**132.43 ± 14.61**	**1082.52 ± 59.35**	**281.11 ± 39.34**	**73.45 ± 5.37**	**58.81 ± 5.36**	310.79 ± 19.25	133.79 ± 15.83
* P. emblica*	A	96.09 ± 8.93	116.21 ± 14.75	960.98 ± 64.11	199.85 ± 27.74	93.88 ± 12.46	75.44 ± 7.37	**523.34 ± 25.46**	**229.45 ± 15.83**
	B	**248.14 ± 11.57**	**261.83 ± 18.42**	**2186.58 ± 97.21**	288.25 ± 48.64	107.61 ± 15.37	87.84 ± 9.41	491.96 ± 23.73	202.08 ± 20.84
	C	176.37 ± 16.73	189.00 ± 21.67	1885.91 ± 75.62	**495.46 ± 45.46**	**155.26 ± 9.36**	**137.53 ± 8.62**	459.33 ± 21.47	181.33 ± 12.48
* T. cordifolia*	A	87.41 ± 9.23	96.65 ± 18.46	801.23 ± 135.26	181.61 ± 35.63	51.47 ± 8.36	36.67 ± 5.71	**353.17 ± 17.38**	**155.87 ± 22.15**
	B	95.52 ± 8.46	109.57 ± 11.48	900.84 ± 126.53	238.43 ± 46.28	58.21 ± 6.83	45.47 ± 6.15	320.96 ± 15.23	134.76 ± 13.55
	C	**106.96 ± 11.39**	**123.13 ± 10.94**	**1033.03 ± 79.47**	**266.25 ± 35.21**	**72.64 ± 5.48**	**55.91 ± 4.82**	309.33 ± 12.26	118.43 ± 14.75

### Effect of herbal extracts on wheat seedling: germination percentage, seedling length, and seedling weight

The process of germination starts by the quiescent dry seeds absorbing water and ending with the lengthening of the embryonic axis^27^. Our study revealed that all herbal extract treatment enhanced the germination rate, germination percentage, seedling length and seedling weight of wheat sprouts (Table 2). Lower concentrations **(**solution A and B) of all treatment showed 100% germination (similar to control), however higher concentrations **(**solution C) of some herbal extracts showed inhibitory character, demonstrating lower germination rate (Table 2). Seedling length and weight also increased with herbal extract treatment; however higher concentrations of some extracts were found to decrease length. The solution C of *Phyllanthus emblica* L. showed maximum increase in seedling length and seedling weight, followed by solution B of *Plumbago zeylanica* L. and solution A of *Bacopa monnieri* L., with 2.33-fold, 2.20-fold and 2.0-fold increase in length and 5.0-fold, 4.57-fold and 3.91-fold increase in seedling weight respectively as compared to control (Fig. 1A,B). It may be concluded that the rate of metabolism was accelerated by seed priming, hastening germination and emergence of seedling. Panuccioa et al.^28^ discovered that *Zea mays* L. (maize) seeds treated with *Rosmarinus officinalis* or *Artemisia* leaf extracts had higher germination percentage. Similarly, priming tomato seeds with *Vinca, Chlorophytum* and *Azadirachta* has been shown to reduce mortality and increase seedling vigour^29^. Herbal plants contain bioactive compounds, such as phytohormones, growth regulators, phenolic acid^30^, which might have played a significant role in regulating the metabolism associated with seed germination and seedling establishment. Our research showed that while a particular concentration of herbal extract stimulated growth, excess of this dose reduced it. Literature reports have documented a negative effect of higher concentration of biostimulant on germination rate^23,25^.

### Effect of treatment on wheatgrass length and fresh biomass

Wheatgrass is a term used to describe wheat germinated during a period of 6–10 days. Wheatgrass has recently gained interest as a health food in various countries and consumed in the form of a ready-made beverage or pill^31^. Wheatgrass is thought to be a good source of vitamins, antioxidants, and minerals^32^. We observed in our experiment that after seed treatment, there was an increase in plant biomass due to enhanced growth and increased height, however, reduction was seen in many of the highly concentrated herbal extract treatment (Table 2). Among the tested 25 extract, highest gain in wheatgrass length and biomass were found in *P. emblica* (1.29-fold and 2.08-fold) followed by *P. zeylanica* (1.28-fold and 2-fold) and *B. monnieri* (1.20-fold and 1.45-fold) than control, whereas *Rauvolfia serpentina* L., *Withania somnifera* L. and *Syzygium aromaticum* L. were least effective treatments (Fig. 1C,D). Some plant-based extracts have recorded improved growth and biomass accumulation in crops after their treatment; such as, moringa leaf extract improved wheat growth and productivity^15^, foliar spraying with seaweed and licorice root extracts improved the vegetative growth of onion seedlings^33^. Similarly, the application of Alfalfa derived protein hydrolysates improved tomato biomass and quality^34^.

### Effect of treatment on antioxidant activity

Through a process known as oxidative stress, environment has a harmful effect on the body^35^. Numerous diseases, including cancer, arthritis, ageing, autoimmune disease, cataract, cardiovascular and neurological disorders are greatly influenced by oxidative stress^36–38^. The presence of substances that can shield a biological system from the detrimental effects of oxidative stress is connected to the antioxidant capability. The increase of protective molecules in the form of antioxidant compounds was another finding from our study. The ability of DPPH to scavenge free radicals and the ability of the FRAP reagent to reduce them were used to assess the antioxidant potential of wheatgrass. In comparison to the control, each treatment considerably increased the antioxidant potential; either through a higher or a lower concentration dose (Table 3). Moderately concentrated leaf extract of *P. emblica* (Solution B) was effective and showed the maximum increase by FRAP (1.94-fold) and DPPH (3.11-fold) method, followed by leaf extract of *P. zeylanica* and *B. monnieri* (Solution C) causing a rise of 1.93-fold and 1.82-fold by FRAP and 2.28-fold and 2.38-fold by DPPH method, respectively (Fig. 2A,B). Exogenous application of biostimulant has reportedly been shown to activate both enzymatic and non-enzymatic antioxidant defence mechanisms in plants^25,26^. Dawoud^39^ discovered a rise in DPPH and FRAP levels and came to the conclusion that *Punica granatum* L. peel extract contains biostimulants that could improve health-promoting metabolites in *B. oleraceae* sprouts. The antioxidant and health-promoting qualities of kidney bean sprouts have also been enhanced by exogenous treatment with natural elicitors^40^. In *Solanum melongena* L., Ali et al.^41^ showed that the antioxidant system can be modified in response to the priming effect of an aqueous garlic extract.

### Effect of treatment on phenolic content

Due to its high antioxidant activity, wheatgrass juice is renowned as a healthful beverage. Ferulic acid, caffeic acid, p-coumaric acid, gallic acid, and syringic acid are the principal phenolic chemicals present in wheatgrass^42^. The flavonoid apigenin, which has potential anti-carcinogenic and anti-oxidant properties, is notably abundant in wheatgrass^43^. By doing this investigation, we were able to increase the total phenolic and flavonoid contents of wheatgrass. Some of the tested extracts displayed multifold increase while others caused mild changes in phenolic levels (Table 3). *P. emblica* extracts exhibited 2.32-fold phenol and 2.43-fold flavonoid, *P. zeylanica* showed 2.26-fold phenol and 2.38-fold flavonoids and *B. monnieri* manifested 2.02-fold phenol and 0.88-fold flavonoid increase while extracts of *R. serpentina W. somnifera* and *S. aromaticum* were least effective (Fig. 2C,D). According to previously published study, priming with biostimulants have improved the levels of phenols, flavonoids, and antioxidant systems in wheat and rice crops^25,44,45^. Using leaf extracts from *Typha angustifolia*, Ghezal et. al.^46^ discovered that the secondary metabolites, including total phenolic and total flavonoid in pea (*Pisum sativum* L*.*) was increased.

### Effect of treatment on enzyme activity

An essential antioxidant enzyme that protects plant against oxidative stress is peroxidases. They catalyze detoxification of hydrogen peroxide and convert them into water^22^. Increase in POD activity by exogenous application of chemical biostimulant had been observed in several studies^23,26^. Similarly, in many plant species, the induction of PAL enzyme activity by chemical elicitors is associated with the build-up of phenolic chemicals^47^. Therefore, an experiment was created to investigate the induction of POD and PAL activity by herbal extracts. An increase in enzyme activity was observed in wheatgrass after treatment, although variation in effect was reflected within treatments (Table 3) The extracts from *P.emblica, P. zeylanica*, and *B. monnieri* were the ones with the highest POD activity (4.11-fold, 3.93-fold and 3.64-fold respectively) while extracts of *R. serpentina* (1.25-fold), *W. somnifera* (1.29-fold), and *S. aromaticum* (1.33 fold) were the least effective one (Fig. 2E). Similarly, the highest PAL activities were shown by the extracts from *B. monnieri, P. zeylanica*, and *P. emblica*, which were 5.35-fold, 4.34-fold, and 3.53-fold, respectively and the lowest PAL activities were shown by the extracts from *R. serpentina*, *W. somnifera*, and *S. aromaticum,* which were 1.49-fold, 1.49-fold, and 1.52-fold respectively (Fig. 2F).

### Effect of treatment on photosynthetic pigments

Chlorophyll has long been recognised as a source of energy for plant metabolism. The chlorophyll concentration of the cell influences photosynthetic effectiveness^43^. In literature reports, the benefits of wheatgrass juice have been linked to its high chlorophyll concentration. Animal models used in scientific research on the health benefits of chlorophyll have demonstrated anti-cancer effects^48^. According to earlier studies, seed priming with a variety of naturally occurring chemicals aided in the enhancement of photosynthetic pigments. An increase in the anthocyanin and chlorophyll content in seeds primed with biostimulants was reported in rice seedlings^49^. The priming of mung bean plant with natural compounds helped them to cope with a number of abiotic challenges, by improving the photosynthesis process and increasing the photosynthetic pigments^50^. However, in our experiment it was observed that herbal extract treatments were less efficient in modulating the total amount of chlorophyll and carotenoids as compared with other tested parameters. The majority of treatments were found to have a non-significant difference in pigment content as compared with the control (Table 3). Pigment level was inversely connected with treatments of highly concentrated herbal extracts and correlated favourably with treatments of lesser concentration. The increase in total chlorophyll was highest in *P. emblica* (2.05-fold), *P. zeylanica* (1.99-fold), and *B. monnieri* (1.97-fold) (Fig. 2G). Carotenoid concentration was also found to be positively connected with treatments using less concentrated herbal extracts and adversely correlated with highly concentration treatments (Table 3). Like chlorophyll, a small number of therapies exhibited significant differences, with highest carotenoid percentage rise observed in *P. emblica* (0.66-fold), *P. zeylanica* (0.64-fold) and *B. monnieri* (0.61-fold) treatments (Fig. 2H).

### Principal Component Analysis (PCA)

**Figure 3:**
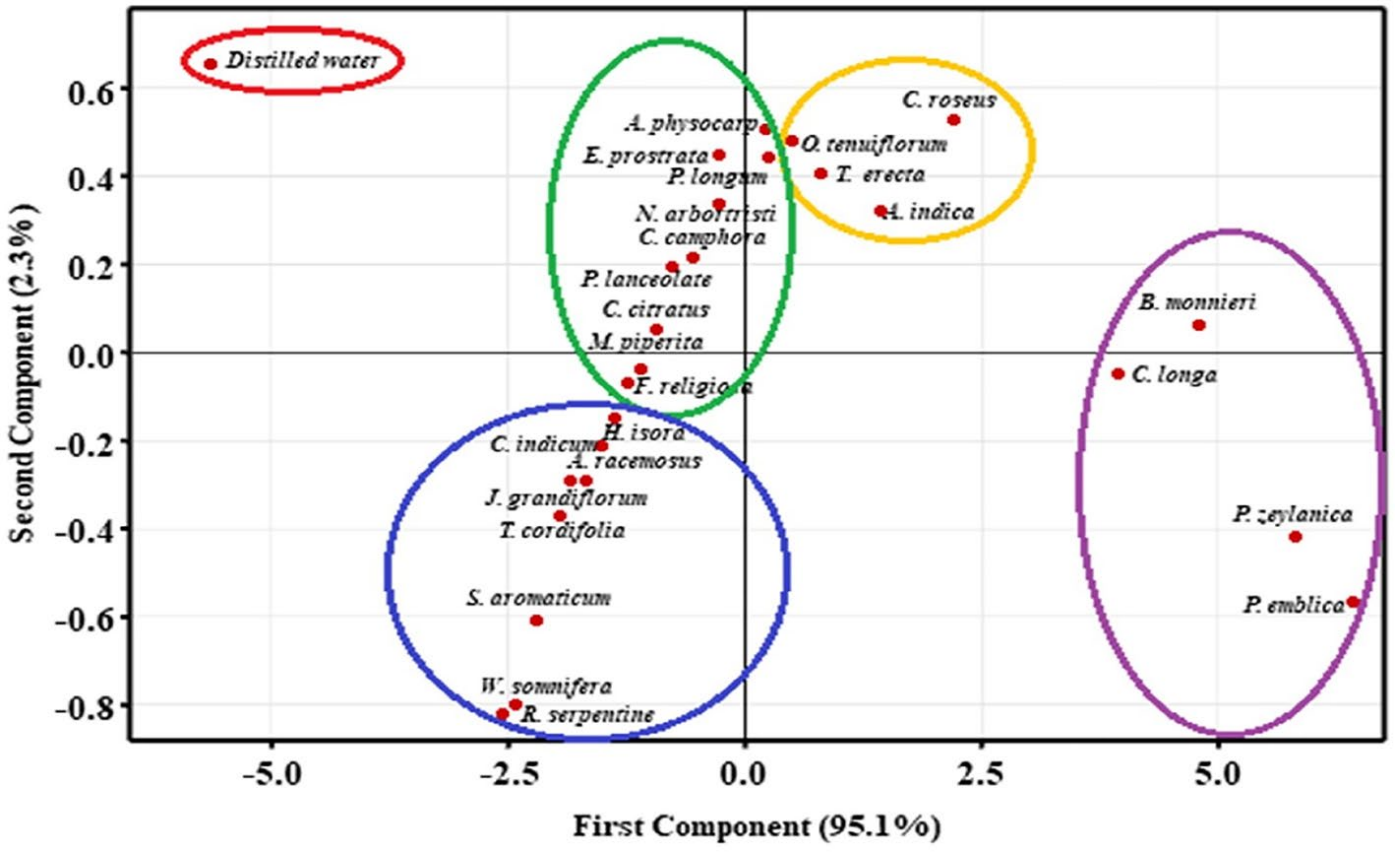
Principal component analysis: Score plot dividing herbal extract into groups based on their effectiveness.

Finding patterns and correlations in data is the major objective of a PCA analysis^51^. The first two primary components were used to produce the score plot, and accounted for 97.4% of the total variance. PCA provided us with a macroscopic categorization that allowed us to separate the herbal samples according to how well they may cause alterations. From the score plot, we could find that all the 25 samples can be clustered into four groups (Fig. 3). Out of these groups, the purple circle, comprising *P. emblica, P. zeylanica, C. roseus*, and *B. monnieri* have the greatest potential to stimulate secondary metabolite production in wheatgrass, followed by *C. roseus, O. tenuiflorum, A. indica*, and *T. erecta*, which are circled in yellow. Green circle indicates that *A. physocarpa*, *E. prostrata, P. longum, N. arbortristis, C. camphora, P. lanceolate, C. citratus, M. piperita,* and *F. religiosa* are moderately active to boost secondary metabolites synthesis in wheatgrass and the blue circle indicates that the extract of *H. isora, C. indicum, A. racemosus, J. Grandiflorum, T. cordifolia, S. aromaticum, W. somnifera*, and *R. serpentine* are least effective in inducing changes (Fig. 3).

**Figure 4:**
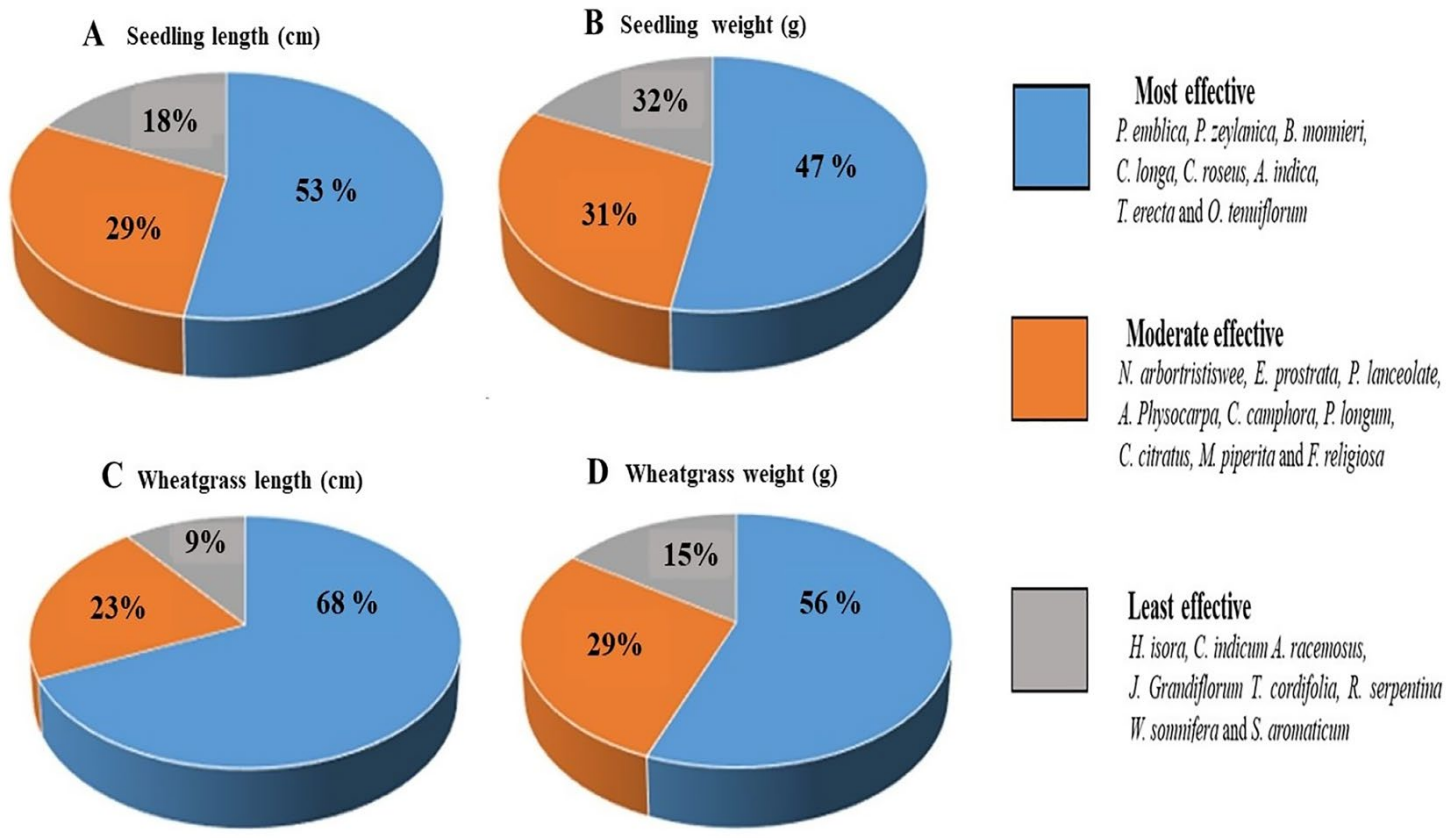
Graphical representation showing quantitative effectiveness of herbal extract groups (in percentage) on morphological parameters. (**A**) Seedling length, (**B**) Seedling weight (**C**) Wheatgrass length (**D**) Wheatgrass weight.

**Figure 5:**
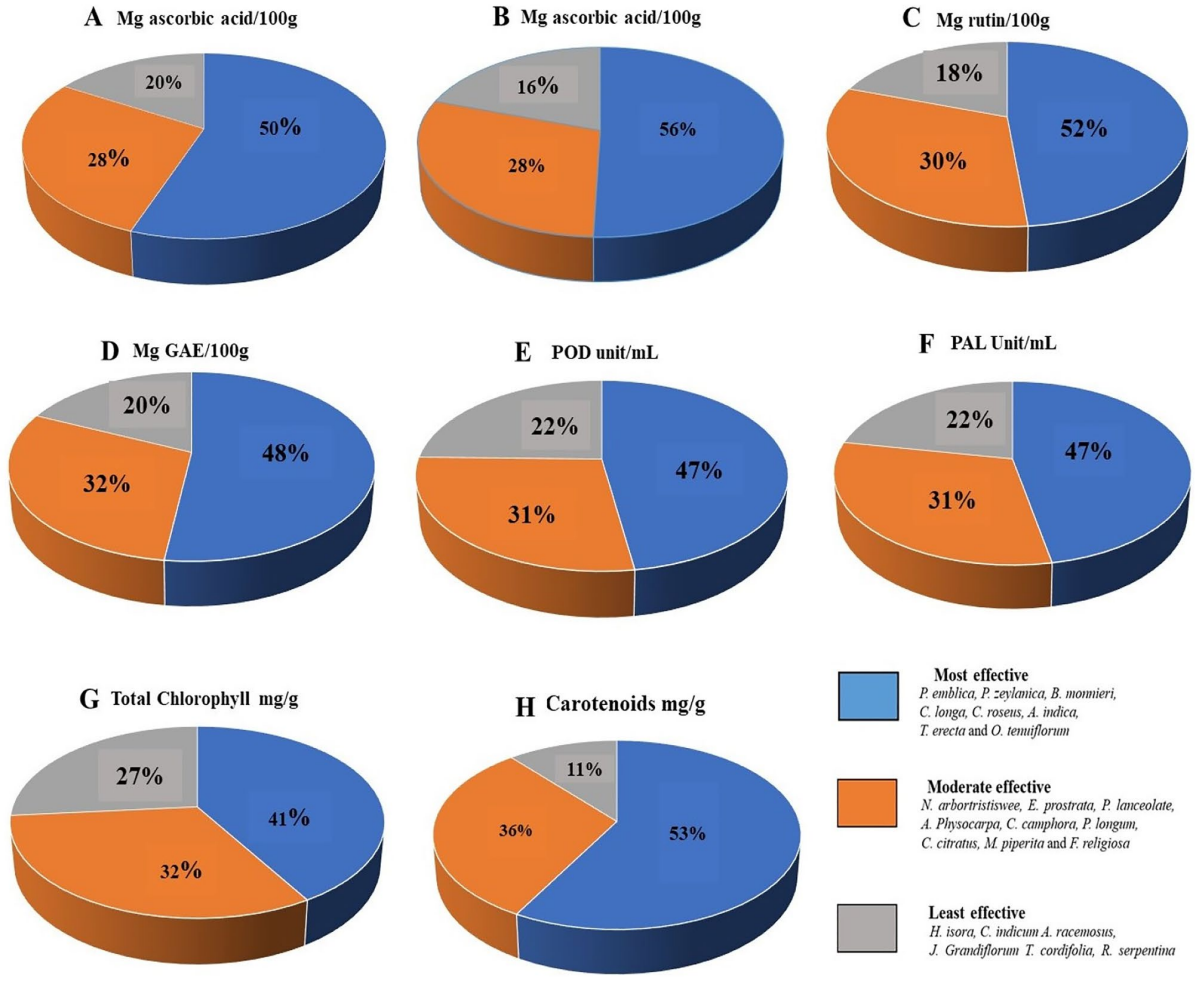
Graphical representation showing quantitative effectiveness of herbal extract groups (in percentage) on biochemical parameters (**A**) DPPH assay, (**B**) FRAP assay, (**C**) Total phenolic, (**D**) Total flavonoids, (**E**) POD activity, (**F**) PAL activity, (**G**) Total chlorophyll and (**H**) Carotenoids.

Results of the PCA analysis clearly shows a variation in biostimulating activity between herbal samples. Next, we were interested to quantify the effectiveness of the extracts based on their activity. Therefore, we divided these 25 herbal samples into three groups as most effective, moderate effective and least effective (each group comprising around eight samples) and calculated their overall contribution in enhancing the growth characteristics. The most effective herbal extract is shown in the blue area of the pie chart and covers around 47–68% of morphological parameters (Fig. 4) and 47–56% of biochemical parameters (Fig. 5). The moderately effective herbal extract percentage is depicted in the orange area of the pie chart and accounts for 23–36% of tested parameters, whereas the least effective herbal extract percentage (grey area) ranges between 9 and 27% (Figs. 4 and 5). The result shows a variation in the activity of the tested herbal samples; where some of the extracts are causing multi-fold increase in growth parameters, while some are completely ineffective. This variation depends on the chemical composition of the extracts and the concentration of their bioactive compounds.

## Conclusion

Natural compounds in agriculture are increasingly popular due to the environmental impact of synthetic chemicals and rising demand for organic foods. This study highlights the potential of various herbal extracts as natural biostimulants, showing their positive effects on wheat germination, biomass, and antioxidant activities. The effectiveness of the herbal extracts varies with the plant type and concentration, indicating a need for future research to identify and characterize these bioactive compounds for wider agricultural applications. This aligns with the current trend towards sustainable agriculture, where natural growth stimulants play a crucial role.

## Data Availability

All data generated or analysed during this study are included in this published article.
